# MtDNA population variation in Myalgic encephalomyelitis/Chronic fatigue syndrome in two populations: a study of mildly deleterious variants

**DOI:** 10.1038/s41598-019-39060-1

**Published:** 2019-02-27

**Authors:** Marianne Venter, Cara Tomas, Ilse S. Pienaar, Victoria Strassheim, Elardus Erasmus, Wan-Fai Ng, Neil Howell, Julia L. Newton, Francois H. Van der Westhuizen, Joanna L. Elson

**Affiliations:** 1 Human Metabolomics, North-West University, Potchefstroom, South Africa; 2 Institute of Cellular Medicine & NIHR Biomedical Research Centre in Ageing and Chronic Disease, Newcastle University, Newcastle-upon-Tyne, United Kingdom; 3 School of Life Sciences, University of Sussex, Falmer, BN1 9PH United Kingdom; 4 Centre for Neuroinflammation and Neurodegeneration, Imperial College London, London, United Kingdom; 5 Newcastle upon Tyne Hospitals NHS Foundation Trust, Newcastle, United Kingdom; 6 Department of Radiation Therapy, UTMB, Galveston, Texas USA; 7 Institute of Genetic Medicine, Newcastle University, Newcastle-upon-Tyne, United Kingdom

## Abstract

Myalgic Encephalomyelitis (ME), also known as Chronic Fatigue Syndrome (CFS) is a debilitating condition. There is growing interest in a possible etiologic or pathogenic role of mitochondrial dysfunction and mitochondrial DNA (mtDNA) variation in ME/CFS. Supporting such a link, fatigue is common and often severe in patients with mitochondrial disease. We investigate the role of mtDNA variation in ME/CFS. No proven pathogenic mtDNA mutations were found. We then investigated population variation. Two cohorts were analysed, one from the UK (n = 89 moderately affected; 29 severely affected) and the other from South Africa (n = 143 moderately affected). For both cohorts, ME/CFS patients had an excess of individuals without a mildly deleterious population variant. The differences in population variation might reflect a mechanism important to the pathophysiology of ME/CFS.

## Introduction

Myalgic encephalomyelitis (ME), also known as chronic fatigue syndrome (CFS) is an illness characterized by post-exertional malaise, cognitive dysfunction, unrefreshing sleep, and other symptoms^1^. Severe fatigue is the defining clinical problem, and sleep or rest does not alleviate this fatigue. ME/CFS is a disease associated with substantial levels of disability in many patients, with a high social and economic cost^2^. Skeletal muscle fatigue, and more specifically post-exertional malaise, is a key feature of ME/CFS, and recent studies point to abnormalities of muscle function^3^. Patients suffering from ME/CFS show a reduced capacity to recover from acidosis on repeat exercise^3,4^, which is suggestive of a role for mitochondrial dysfunction in the pathophysiology of this disease. *In vitro* investigations have supported this hypothesis with studies showing functional mitochondrial abnormalities such as a lower mitochondrial respiratory function in ME/CFS patients^5^.

ME/CFS is clinically heterogeneous with the patients most severely affected being house- or even bed-bound for months and sometimes years. Recent findings have indicated that the house-bound group represents one quarter of the patient population^2^. The functional abilities of these patients are significantly more impaired in many aspects compared to ME/CFS patients who were not house-bound, including but not limited to fatigue, post-exertional malaise, sleep and pain, as well as neurocognitive, autonomic, neuroendocrine, and immune functioning^2^. However, the factors that make a patient less or more likely to fall into this severely affected group are unknown, as are the factors linked to recovery. Evidence suggests that recovery remains rare in the ME/CFS patient population regardless of disease severity^6^.

Clinically proven pathogenic mtDNA mutations are recognized as a cause of maternally-inherited disorders, with a minimum prevalence rate of 1 mutation in 5,000 (20 per 100,000) people^7^. Such mtDNA mutations frequently cause multisystem disorders with fatigue being prevalent in these patient groups^8^. Hundreds or even thousand of copies of mtDNA are present in a /cell, this is linked to the energetic demands of the cell. These copies can either be identical, a state called homoplasmy, or two or more species of mtDNA can be present in a state referred to as heteroplasmy. Thus, a possible approach is to investigate whether mtDNA mutations are present either at heteroplasmy levels sufficient to cause mtDNA disease or at sub-clinical levels that are too low to cause primary mtDNA disease, but are perhaps at sufficient levels to act as a risk factor or affect the course of a complex disease such as ME/CFS.

Beyond such recognised pathogenic mtDNA mutations, many studies have suggested a role for common mtDNA variants in complex diseases, with mtDNA variants either modulating susceptibility to a disease and/or affecting the course of the disease, including those where fatigue is an important feature of disease^9,10^. While many studies have reported a significant association of specific mtDNA haplogroups with a number of complex disorders, there is often substantial disagreement among different studies examining the same phenotype.

Another possibility is that rare mtDNA population variants might have a role in the disease process, since rare variants are predicted to be more mildly deleterious as such variants are removed by purifying selection over generations^11^. This is supported by recent works^12,13^, using a computational tool, MutPred, that has been widely used in the mitochondrial context^14^. Given this it might be expected to see a greater number of rare/mildly deleterious variants in any given patient group^15^, which may implicate a role in the disease process.

In addition to acting as a susceptibility factor to disease, it has also been suggested that mtDNA variants might modify the course of common complex diseases^16^. In line with this a recent study of 193 ME/CFS patients and 196 age- and gender-matched controls, reported haplogroups J, U and H, as well as eight mtDNA SNPs are significantly associated with particular ME/CFS symptoms in patients (course of disease), but not with increased susceptibility to ME/CFS (onset of disease)^10^.

In the current study, using mtDNA sequence data from ME/CFS patients from both the United Kingdom (UK) and South Africa (RSA), we ask whether mtDNA population variants alter susceptibility to ME/CFS. Due to the maternal inheritance pattern of mtDNA, linking mtDNA variation to common complex traits has been recognized as a difficult task, with models used in nuclear association genetics proving unsuitable^17,18^. There have been a number of improvements in the design of haplogroup association studies over the last 10 years, such as the importance of a replicate cohort^ being recognized15,19^. However, there are still many doubts as to whether this simple methodology is the correct approach to assess the role of mtDNA variation in complex disorders. Indeed, a number of prior studies have applied the haplogroup association approach in complex diseases where fatigue is an important clinical feature, such as multiple sclerosis^9,20^, but the results were inconclusive. Here, we applied an improved approach which focuses on variants predicted computationally to be mildly deleterious, most of which are rare. This approach takes advantage of the advances in bioinformatics^21,22^ using the mtDNA-server^23^ and MutPred tools^14,23^.

## Results

### Heteroplasmy analysis

To investigate the possibility that ME/CFS patients harbour clinically proven mtDNA mutations, either above the threshold required for mtDNA disease or at a sub-threshold level, the complete mitochondrial genomes of the (n = 89 moderate; 29 severely affected) patients from the UK and (n = 143) South African ME/CFS patients were analysed. Only two mutations associated with clinically proven mitochondrial disease were seen, m.3337G>A^24^ in a patient and m.11778G>A^25^ in a control participant, both in the South African cohort. However, these mutations were heteroplasmic at the 5% and 16.5% level respectively, and as a result these mutations are unlikely to have a phenotypic role. No other clinically proven mtDNA mutations were detected in any of the patient or control groups. It should be noted that such frequencies of low level pathogenic mtDNA mutations are entirely consistent with large population studies considering this question^26^, as well as prior studies on this phenotype^10,27^.

### Haplogroup distributions

Human mtDNAs can be assigned to one of several haplogroups. This traditional classification system was originally based on the presence or absence of one or a small number of likely benign polymorphisms^28^, rather than mutations with functional consequences. However, the advent of large-scale sequencing has led to the identification of a vast number of haplogroup-specific polymorphisms, allowing haplogroup classifications to be expanded into more and more subgroups^29^. To put our analysis into the context of prior studies, a simple haplogroup distribution analysis was performed.

To ensure a robust comparison between the groups from the UK and the RSA, only the frequencies of the nine haplogroups of European origin (HVUKTJIXW) from the RSA ME/CFS cohort were used, as the group from the UK (North East England) was of lower diversity. The haplogroup distributions for the UK and RSA cohorts – ME/CFS and control - are given in Table 1. Comparing the haplogroup distributions between the UK ME/CFS patients and controls with a Monte Carlo-based approach, no significant difference was observed (ns, *p* = 0.31). Similarly, there was no significant difference in haplogroup distribution between RSA patients and controls (ns, *p* = 0.55). Thus as in the work of Billing-Ross *et al*., mtDNA haplogroup was not seen to affect the susceptibility to ME/CFS^10^.

**Table 1:** Haplogroup distribution for each group in the two cohorts.

*Haplogroups*	*Number of persons assigned to each haplogroup per group (% in brackets)*				
	*UK*			*RSA*	
	*ME/CFS severe*	*ME/CFS patients*	*Controls*	*ME/CFS patients*	*Controls*
	*(N* = *29)*	*(N* = *89)*	*(N* = *64)*	*(N* = *143)*	*(N* = *98)*
HV	14 (48)	41 (46)	22 (34)	66 (34)	37 (38)
I	—	3 (3)	2 (3)	1 (0.5)	1 (1)
J	5 (17)	12 (14)	13 (20)	15 (8)	11 (11)
K	1 (3)	5 (6)	9 (14)	12 (6)	8 (8)
R	—	—	—	1 (0.5)	5 (5)
T	3 (10)	11 (12)	5 (8)	13 (7)	14 (14)
U	5 (17)	13 (15)	11 (17)	32 (17)	20 (20)
W	—	2 (2)	1 (2)	1 (0.5)	2 (2)
X	1 (3)	—	1 (2)	2 (1)	—

UK: United Kingdom; RSA: Republic of South Africa; ME/CFS: Myalgic encephalomyelitis/chronic fatigue syndrome.

### Network analysis

**Figure 1:**
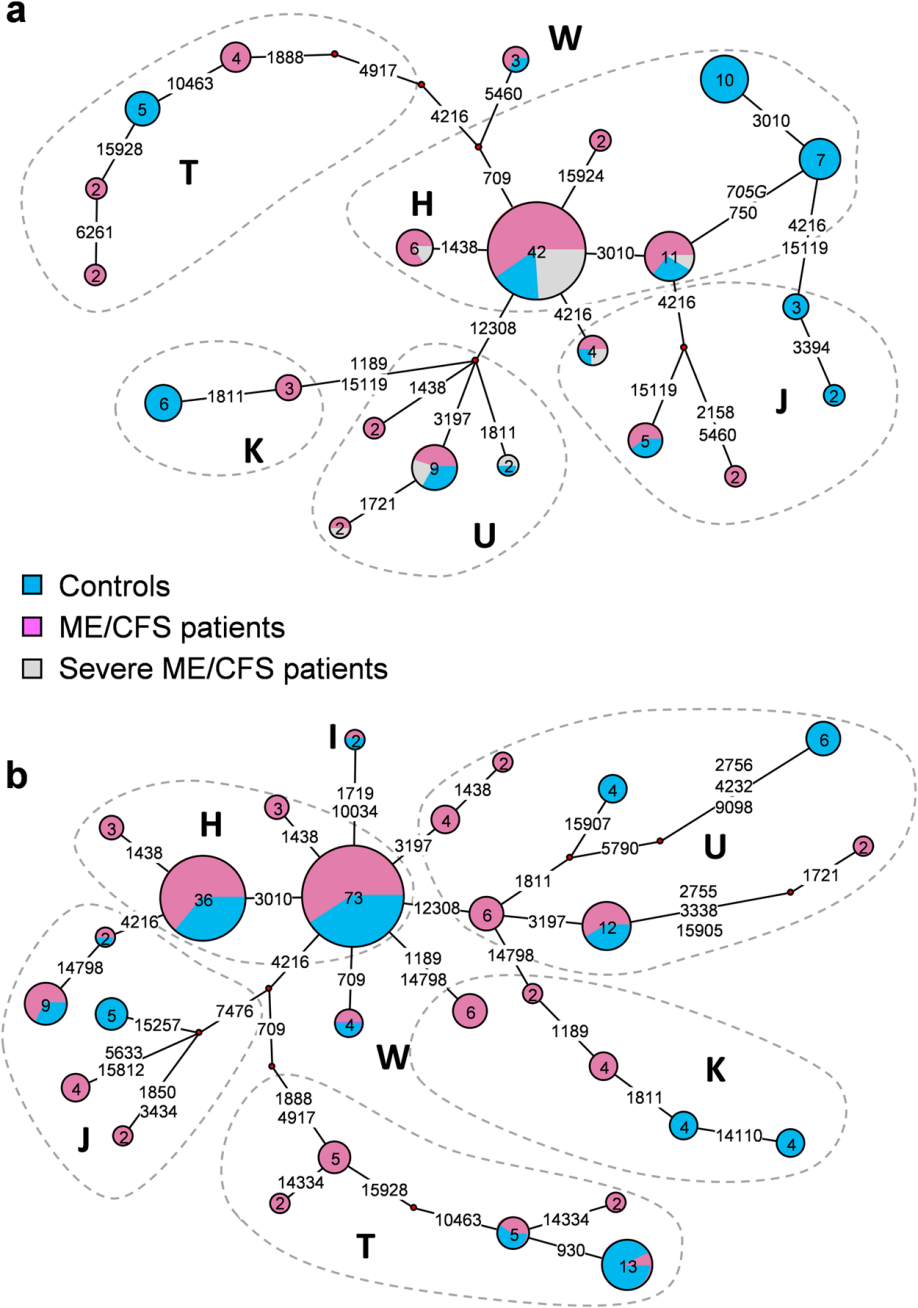
Shows functional networks of UK participants (**a**) and RSA (**a**) participants. Ellipses indicate clusters of branches and nodes that fall within similar haplogroups. Small red nodes indicate potential median vectors. Pie-chart nodes indicate the proportion of individuals per phenotype classification, as well as the number of participants per node. mtDNA variants responsible for links are listed. Transversions are shown in italics.

Functional networks were produced for the UK cohort (Fig. 1a) and the RSA cohort (Fig. 1a). These networks represent all possible least complex phylogenetics trees, based on the variants included as described in Methods. Sequences that are very similar cluster together in smaller or larger nodes. Nodes are ordered along a phylogenetic tree with links indicating the variants by which a connected node deviated from another^30^. In both cohorts, controls were more abundant in nodes that were separated by several variants from the central, haplogroup H-dominated nodes. These peripheral nodes frequently contained mtDNAs that could be assigned to haplogroups T, K and U, although as noted in the previous section, no significant haplogroup associations were found.

### Individuals with a potentially mildly deleterious mtDNA variant

Haplogroup association studies are confounded by a number of factors, including population stratification, with this and other factors resulting in elevated type 1 error^18,31^. These limitations, coupled with the relatively low statistical power of haplogroup association studies^32^, underscore the fact that new methods are required for discerning the pathogenic and/or etiological role of mtDNA mutations in a common complex disorder^21,22^. We determined the number of individuals with mtDNA sequences containing a variant with a MutPred score over the 0.5 threshold, such variants being considered “actionable hypothesis” or candidates for having a functional effect^14^, here referred to as “mildly deleterious”.

**Figure 2:**
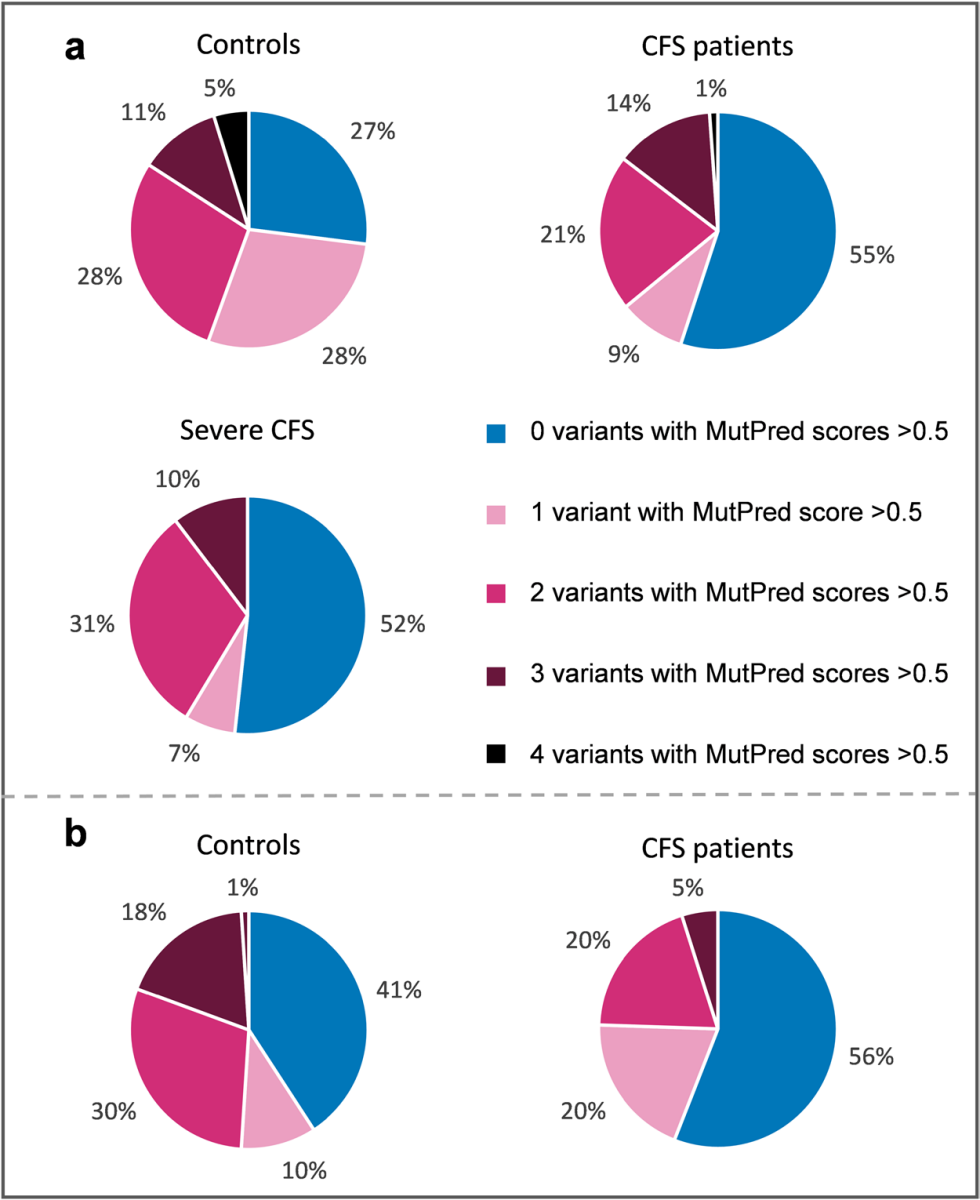
Pie-charts indicating the percentage of controls and ME/CFS patients that harbour variants with MutPred scores above 0.5 for the (**a**) UK cohort and (**b**) South African cohort. ME/CFS: Myalgic encephalomyelitis/chronic fatigue syndrome.

The number of controls and patients in each population that harbours none, one or more mildly deleterious variants are summarised in Table 2. Figure 2 illustrates these numbers as percentages of each group.

**Table 2:** Number of individuals that harbour high MutPred scored variants.

*Number of variants with MutPred scores above 0*.*5*	*Number of individuals harbouring variants with MutPred scores >0*.*5 (% in brackets)*				
	*UK*			*RSA*	
	*Controls*	*ME/CFS patients*	*severe ME/CFS*	*Controls*	*ME/CFS patients*
	*(N* = *64)*	*(N* = *89)*	*(N* = *29)*	*(N* = *98)*	*(N* = *143)*
0	17 (27)	49 (55)	15 (52)	40 (41)	80 (56)
1	18 (28)	8 (9)	2 (7)	10 (10)	28 (20)
2	18 (28)	19 (21)	9 (31)	29 (30)	28 (20)
3	7 (11)	12 (14)	3 (10)	18 (18)	7 (5)
4	3 (5)	1 (1)	—	1 (1)	—

UK: United Kingdom; RSA: Republic of South Africa; ME/CFS: Myalgic encephalomyelitis/chronic fatigue syndrome.

For the cohorts collected in the North East of England, 49 (55%) of the moderate ME/CFS patients were without a mildly deleterious variant. Comparing this result to the controls from the United Kingdom, only 17 (27%) of these individuals had no such variants. Conversely 45% of moderate ME/CFS patients had one or more mildly deleterious variants, compared to 72% of controls from the UK who harboured one or more such variants (see Fig. 2). These differences were found to be highly significant (*p* = 0.0008) with a Fisher’s Exact test. This observation suggests that individuals with ME/CFS are *less* likely to have an mtDNA variant that is predicted to have a functional impact.

As mentioned above, as many as 25% of those with ME/CFS are severely affected, being house or even bed bound. A cohort of such patients was recruited in the North East of England (UK). Of the 29 severe ME/CFS patients 15 (52%) were without a mildly deleterious variant. Comparing these numbers with those of UK controls using a Fisher’s Exact test, a significant difference (*p* = 0.03) was found. Thus even the most severely affected patients had *fewer* mildly deleterious variants than the controls. It is important to note that when the moderately and severely affected patients are compared using a Fishers exact test no difference is observed (*p* = 0.83). The analysis was repeated in the RSA cohort, including only those ME/CFS cases that fall within the nine haplogroups designated as European (HVUKTJIXW) to ensure that differences in lineage diversity could not impact upon the results. Conducting the same analysis with the ME/CFS 143 patients from the RSA, there were 80 (56%) individuals with predicted mildly deleterious variants; considering the healthy controls from the RSA, 40 (41%) individuals had no such variants. That leaves 59% of controls from South Africa compared to only 44% of ME/CFS patients who harbour one or more mildly deleterious mtDNA variants. A Fisher’s exact test again found these differences to be statistically significant (*p* = 0.03). Our analyses thus show, for both UK and RSA cohorts, those with ME/CFS have *fewer* mildly deleterious variants than controls.

## Discussion

We have investigated a possible role of mtDNA variants in ME/CFS. This was done firstly by reviewing the sequence data of each individual for the presence of clinically proven pathogenic mtDNA mutations associated with primary mitochondrial disease. We ruled out the possibility that previously identified pathogenic mtDNA mutations are contributing to ME/CFS, a result that was not unexpected^10,27^.

Secondly, we considered the possible role of population variants in ME/CFS, with a focus on variants that are predicted to be mildly deleterious by computational methods. These variants are frequently rare at the population level resulting in an absence of population stratification, and thus in a lower chance of false association or type 1 error. Such false associations are believed to be common in mtDNA association studies applying the haplogroup association model^18^. The current approach has been utilized in two prior studies^21,22^ that considered Alzheimer and cardiovascular disease respectively.

We compared the number of individuals in both the ME/CFS patient and control groups with variants classified as mildly deleterious, to the number of individuals without such variants. In both the UK and RSA cohorts, there was a significant difference between the patients and the controls, with the ME/CFS patients having a higher percentage of patients *without* a variant predicted to be mildly deleterious. Although surprising, this observation was seen in two independent cohorts, suggesting that these differences are not due to chance. One of the reasons for this observation might have been that deleterious variants confer disease susceptibility in only severely affected ME/CFS patients or modulate fatigue severity among ME/CFS patients. However, when comparing patients that were severely affected with controls in the UK cohort, again ME/CFS patients had fewer variants predicted to be mildly deleterious. Furthermore, there was no difference in the number of patients with such variants between the moderate and severely affected groups. Taken together, these data suggest that our observation is not the result of a simple patient stratification effect, although additional replication cohorts of moderate and severely affected ME/CFS cohorts are needed to confirm this.

It has been proposed that those with ME/CFS might not have a problem with ATP production^33^, but rather with ATP utilization. Therefore, we consider this genetic difference in mtDNA variation as an accurate observation which may prove to have a biological relationship to the function and regulation of the OXPHOS system and subsequent wide-ranging immediate and downstream consequences on energy metabolism^34,35^. It should be considered that the wider homeostasis and/or responsiveness of the various elements of energy metabolism are affected in ME/CFS, rather than merely single “segments” of energy pathways such as ATP production.

A number of mitochondrial abnormalities in ME/CFS have previously been reported, indicating that mitochondrial dysfunction may play a role in the pathogenesis of disease, at least in a sub-set of patients^36^. Previously ME/CFS patients have been shown to have significantly lower mitochondrial function than healthy controls^5^. Detection of these mtDNA variants has the potential to be used as a tool for measuring mitochondrial dysfunction in ME/CFS. Another avenue of investigation in the mitochondrial field is copy number analysis. Mitochondrial DNA copy number (mtDNAcn) has been reported as an indirect representative of mitochondrial function and as a biomarker of disease with studies. As an example, in Parkinson’s disease (PD), mtDNAcn was elevated in the pedunculopontine nucleus (PPN), which is a brainstem region associated with progression of motor and non-motor symptoms of PD^37^. Other neurodegenerative conditions in which alterations in mtDNAcn have been reported include Multiple Sclerosis. The results in this phenotype are conflicting, with some showing evidence for reduced copy number^38^, while others indicate an increase^39^. Additionally. MtDNAcn was shown not to be associated with fatigue status in Primary Sjögren’s Syndrome^30^. Taken together these papers demonstrate that in heterogeneous diseases with a variable course small single time point studies will produce data that is difficult to interpret and is likely to conflict between studies.

In conclusion, this is the first paper to demonstrate *mitochondrial* genetic differences between ME/CFS patients and controls. It also demonstrates the power of mtDNA analysis focused on variants likely to be of a functional effect to detect differences between case and control cohorts where the traditional haplogroup association method frequently fails to do so. Future studies need to include larger cohorts from multiple centres, within and between nations, with standardized sample handling. These studies need to take a multi-disciplinary approach linking genetics, including mtDNA copy number analysis and bioenergetics. Given the changing nature of the disease, longitudinal studies would seem to be essential to further understanding by allowing us to determine how mtDNA varation and mitochondrial dysfunction relates to fluctuations in symptom severity.

## Methods

### Ethical approval and informed consent

This study was conducted in two independent cohorts, one from England and the other from South Africa. All experimental protocols were approved by the corresponding intuitional committees, namely the CRN National Coordinating Centre (CRNCC) | NIHR Clinical Research Network (CRN) in England (UK ME/CFS - IRAS ID 221364) and the Health Research ethics committee (HREC) of the North-West University in South Africa (SABPA: NWU 00036-07-S6, CFS: NWU 00102-12). All study procedures carried out in accordance with relevant guidelines and regulations of Newcastle University and North-West University. All participants gave informed consent and were over the age of 18.

### Patient cohorts

We used two well-characterised cohorts of ME/CFS patients, one from the North East of England (n = 89 moderately affected; 29 severely affected) and the other from South Africa (n = 143 moderately affected). Both cohorts met the Fukuda diagnostic criteria^1^. Potentially confounding causes of fatigue, including depression, were excluded in all patients. The two control cohorts were regionally matched and had been collected for two prior studies. The controls from the North East of England (n = 64) were used previously for a variant load study on Alzheimer’s disease^15^. The control cohort from South Africa (n = 98) was comprised of healthy high school teachers assembled previously for a study on hypertension and diabetes^22^.

### Sequencing

Sequencing of the ME/CFS samples from both the UK and RSA was carried out at Source Bioscience using a fluidigm technology. The sequencing methodology for the controls has been described previously for the UK controls^15^ and for the RSA controls^22^. The reference sequence used in all datasets was the revised Cambridge reference sequence (rCRS).

### Network analysis

After selective pruning of the total mtDNA variants to those in (a) protein-encoding genes with MutPred pathogenicity scores above 0.5, and (b) rRNA and tRNA variants, a functional maximum parsimony (MP) network analysis was performed with the NETWORK (version 5.0.0.3) software package (http://www.fluxus-engineering.com/sharenet.htm). Transversions, being chemically less likely to occur, were weighted three times more than transitions. Star contractions were performed using a maximum radius of 5. This step was followed by reduced median (RM)^40^ processing (reduction threshold *r* was 1). The “Frequency >1” criterion was activated to exclude sequences which are unique to the dataset. Network figures were produced using NETWORK Publisher (version 2.1.1.2), PowerPoint (Microsoft Office 365) and Adobe Photoshop Elements (version 10.0).

### Data analyses

Sequencing data was processed using the online mtDNA-server (mtdna-server.uibk.ac.at)^23^ tool. With this tool, homo- and heteroplasmy variants were identified. The pathogenicity status of heteroplasmic variants were assessed using various clinical and online databanks e.g. MitoMap, and the application of accepted scoring criteria^41,42^. For all other analyses described below, only homoplasmic variants and those with a heteroplasmy level above 90% were used, thus only inherited and not somatic variants were considered. Haplogroups were assigned using the online Haplogrep 2.0 tool (haplogrep.uibk.ac.at)^43^. The control data from the UK is Sanger Sequence data, the processing of which is described in^44^.

### Classification and selection of mtDNA variants, as compared by group

Analysis using variants predicted to be mildly deleterious are less prone to the effects of population stratification because they typically analyse rare variants that are not stratified between geographical locations. The variants are assessed using the MutPred program, which assigns a “pathogenicity” score between 0–1 (an amino acid change with 0 is predicted to be perfectly benign). A score above 0.5 for an amino acid change is classified as an “actionable hypothesis”^14^. Variants with pathogenicity scores below the “actionable hypothesis” threshold (0.5) are considered less likely to be deleterious or to have an impact on protein function; instead, they are more likely to be common population variants. The inclusion of these more numerous but low-scoring, low-impact variants in the analyses could be problematic, especially because the resultant “noise” differs greatly among different population groups^45^. Therefore, as in some of our previous studies, we have not included variants scoring below 0.5 in the current analyses^22^.

### Statistical analyses

All statistical analyses were performed using SPSS Statistics (Version 25), Prism (Version 23) or GraphPad (Version 7). Haplogroup distribution between ME/CFS patient cohorts and their corresponding control were performed using a Monte Carlo based approach, this methodology is part of the standard package but more accurate than the Chi Square estimation. Fisher’s exact tests were utilised to compare the number of patients and controls in each cohort that have mtDNA variants with MutPred scores above 0.5 (mildly deleterious) with those who do not.

## Data Availability

Whole mtDNA sequences for the South African controls have been deposited in the sequence read archive (SRA) of NCBI under BioProject accession number PRJNA403942 (https://www.ncbi.nlm.nih.gov/bioproject/403942). The UK controls can be found https://link.springer.com/article/10.1007%2Fs00439-005-0123-8 as electronic supplemental material.

## References

[1] Fukuda K (1994). The chronic fatigue syndrome: a comprehensive approach to its definition and study. International Chronic Fatigue Syndrome Study Group. Ann Intern Med..

[2] Pendergrast T (2016). Housebound versus nonhousebound patients with myalgic encephalomyelitis and chronic fatigue syndrome. Chronic Illn.

[3] Jones, D. E. *et al*. Loss of capacity to recover from acidosis on repeat exercise in chronic fatigue syndrome: a case-control study. *Eur J Clin Invest*. **42**, 186–194, 10.1111/j.1365-2362.2011.02567.x. Epub02011 Jul 02512 (2012). 10.1111/j.1365-2362.2011.02567.x 21749371

[4] Brown, A. E., Jones, D. E., Walker, M. & Newton, J. L. Abnormalities of AMPK activation and glucose uptake in cultured skeletal muscle cells from individuals with chronic fatigue syndrome. *PLoS One*. **10**, e0122982, 0122910.0121371/journal.pone.0122982. eCollection0122015 (2015). 10.1371/journal.pone.0122982 PMC4383615 25836975

[5] Tomas, C. *et al*. Cellular bioenergetics is impaired in patients with chronic fatigue syndrome. *PLoS One*. **12**, e0186802, 0186810.0181371/journal.pone.0186802. eCollection0182017 (2017). 10.1371/journal.pone.0186802 PMC5655451 29065167

[6] Cairns R, Hotopf M (2005). A systematic review describing the prognosis of chronic fatigue syndrome. Occup Med (Lond)..

[7] Gorman, G. S. *et al*. Prevalence of nuclear and mitochondrial DNA mutations related to adult mitochondrial disease. *Ann Neurol*. **77**, 753–759, 710.1002/ana.24362. Epub22015 Mar 24328 (2015). 10.1002/ana.24362 PMC4737121 25652200

[8] Gorman, G. S. *et al*. Perceived fatigue is highly prevalent and debilitating in patients with mitochondrial disease. Neuromuscul Disord. **25**, 563–566, 10.1016/j.nmd.2015.1003.1001. Epub2015 Apr 1023 (2015). 10.1016/j.nmd.2015.03.001 PMC4502433 26031904

[9] Ban M (2008). Investigation of the role of mitochondrial DNA in multiple sclerosis susceptibility. PLoS One..

[10] Billing-Ross, P. *et al*. Mitochondrial DNA variants correlate with symptoms in myalgic encephalomyelitis/chronic fatigue syndrome. *J Transl Med*. **14****:****19**, 10.1186/s12967-12016-10771-12966 (2016). 10.1186/s12967-016-0771-6 PMC4719218 26791940

[11] Elson, J. L., Turnbull, D. M. & Howell, N. Comparative genomics and the evolution of human mitochondrial DNA: assessing the effects of selection. *Am J Hum Genet*. **74**, 229–238. Epub 2004 Jan 2007 (2004). 10.1086/381505 PMC1181921 14712420

[12] Pereira, L., Soares, P., Radivojac, P., Li, B. & Samuels, D. C. Comparing phylogeny and the predicted pathogenicity of protein variations reveals equal purifying selection across the global human mtDNA diversity. *Am J Hum Genet*. **88**, 433–439, 10.1016/j.ajhg.2011.1003.1006. Epub 2011 Mar1031 (2011). 10.1016/j.ajhg.2011.03.006 PMC3071914 21457906

[13] Soares, P. *et al*. Evaluating purifying selection in the mitochondrial DNA of various mammalian species. *PLoS One* **8**, e58993, 10.51371/journal.pone.0058993. Epub0052013 Mar 0058922 (2013). 10.1371/journal.pone.0058993 PMC3606437 23533597

[14] Li, B. *et al*. Automated inference of molecular mechanisms of disease from amino acid substitutions. *Bioinformatics*. **25**, 2744–2750, 10.1093/bioinformatics/btp2528. Epub2009 Sep 2743 (2009). 10.1093/bioinformatics/btp528 PMC3140805 19734154

[15] Elson, J. L. *et al*. Does the mitochondrial genome play a role in the etiology of Alzheimer’s disease? *Hum Genet*. **119**, 241–254. Epub 2006 Jan 2012 (2006). 10.1007/s00439-005-0123-8 16408223

[16] Bregman JA (2017). Mitochondrial Haplogroups Affect Severity But Not Prevalence of Diabetic Retinopathy. Invest Ophthalmol Vis Sci..

[17] Elson, J. L., Majamaa, K., Howell, N. & Chinnery, P. F. Associating mitochondrial DNA variation with complex traits. *Am J Hum Genet*. **80**, 378–382; author reply 382-373 (2007). 10.1086/511652 PMC1785337 17304709

[18] Salas, A. & Elson, J. L. Mitochondrial DNA as a risk factor for false positives in case-control association studies. *J Genet Genomics*. 42, 169–172, 10.1016/j.jgg.2015.1003.1002. Epub 2015 Mar1017 (2015). 10.1016/j.jgg.2015.03.002 25953355

[19] Chinnery, P. F., Elliott, H. R., Syed, A. & Rothwell, P. M. Mitochondrial DNA haplogroups and risk of transient ischaemic attack and ischaemic stroke: a genetic association study. *Lancet Neurol*. **9**, 498–503, 10.1016/S1474-4422(1010)70083-70081. Epub72010 Mar 70031 (2010). 10.1016/S1474-4422(10)70083-1 PMC2855429 20362514

[20] Yu X (2008). mtDNA nt13708A variant increases the risk of multiple sclerosis. PLoS One..

[21] Pienaar IS, Howell N, Elson JL (2017). MutPred mutational load analysis shows mildly deleterious mitochondrial DNA variants are not more prevalent in Alzheimer’s patients, but may be under-represented in healthy older individuals. Mitochondrion.

[22] Venter, M., Malan, L., van Dyk, E., Elson, J. L. & van der Westhuizen, F. H. Using MutPred derived mtDNA load scores to evaluate mtDNA variation in hypertension and diabetes in a two-population cohort: The SABPA study. *J Genet Genomics*. **44**, 139–149, 10.1016/j.jgg.2016.1012.1003. Epub2016 Dec 1026 (2017). 10.1016/j.jgg.2016.12.003 28298255

[23] Weissensteiner, H. *et al*. mtDNA-Server: next-generation sequencing data analysis of human mitochondrial DNA in the cloud. *Nucleic Acids Res*. **44**, W64–69, 10.1093/nar/gkw1247. Epub2016 Apr 1015 (2016). 10.1093/nar/gkw247 PMC4987870 27084948

[24] Zifa, E. *et al*. A novel G3337A mitochondrial ND1 mutation related to cardiomyopathy co-segregates with tRNALeu(CUN) A12308G and tRNAThr C15946T mutations. *Mitochondrion*. **8**, 229–236, 10.1016/j.mito.2008.1004.1001. Epub2008 May 1027 (2008). 10.1016/j.mito.2008.04.001 18502698

[25] Hudson, G. *et al*. Clinical expression of Leber hereditary optic neuropathy is affected by the mitochondrial DNA-haplogroup background. *Am J Hum Genet*. **81**, 228–233. Epub 2007 Jun 2004 (2007). 10.1086/519394 PMC1950812 17668373

[26] Elliott HR, Samuels DC, Eden JA, Relton CL, Chinnery PF (2008). Pathogenic mitochondrial DNA mutations are common in the general population. Am J Hum Genet..

[27] Schoeman EM (2017). Clinically proven mtDNA mutations are not common in those with chronic fatigue syndrome. BMC Med Genet..

[28] Torroni, A. *et al*. A signal, from human mtDNA, of postglacial recolonization in Europe. *Am J Hum Genet*. **69**, 844–852. Epub 2001 Aug 2021 (2001). 10.1086/323485 PMC1226069 11517423

[29] van Oven M, Kayser M (2009). Updated comprehensive phylogenetic tree of global human mitochondrial DNA variation. Hum Mutat..

[30] De Menezes ECS (2018). Mitochondrial DNA copy number is not associated with fatigue status in Primary Sjögren’s Syndrome. Fatigue: Biomedicine, Health & Behavior.

[31] Herrnstadt, C. & Howell, N. An evolutionary perspective on pathogenic mtDNA mutations: haplogroup associations of clinical disorders. *Mitochondrion*. **4**, 791–798. Epub 2004 Oct 2001 (2004). 10.1016/j.mito.2004.07.041 16120433

[32] Samuels, D. C., Carothers, A. D., Horton, R. & Chinnery, P. F. The power to detect disease associations with mitochondrial DNA haplogroups. *Am J Hum Genet*. **78**, 713–720. Epub 2006 Feb 2017 (2006). 10.1086/502682 PMC1424681 16532401

[33] Lawson N, Hsieh CH, March D, Wang X (2016). Elevated Energy Production in Chronic Fatigue Syndrome Patients. J Nat Sci.

[34] Reinecke, F., Smeitink, J. A. & Van der Westhuizen, F. H. OXPHOS gene expression and control in mitochondrial disorders. *Biochim Biophys Acta*. **1792**, 1113–1121, 10.1016/j.bbadis.2009.1104.1003. Epub 2009 Apr 1121 (2009). 10.1016/j.bbadis.2009.04.003 19389473

[35] Bothma K, van der Westhuizen, F. H. & Louw, R. Metabolomics of Mitochondrial Disease. *Mitochondrion* 97–110 (2017). 10.1016/j.mito.2017.05.012 28576558

[36] Tomas, C. & Newton, J. Metabolic abnormalities in chronic fatigue syndrome/myalgic encephalomyelitis: a mini-review. *Biochem Soc Trans*. **46**, 547–553, 10.1042/BST20170503. Epub 20172018 Apr 20170517 (2018). 10.1042/BST20170503 29666214

[37] Bury, A. G. *et al*. Mitochondrial DNA changes in pedunculopontine cholinergic neurons in Parkinson disease. *Ann Neurol*. **82**, 1016–1021, 10.1002/ana.25099. Epub22017 Dec 25094 (2017). 10.1002/ana.25099 29149768

[38] Lowes H, Pyle A, Duddy M, Hudson G (2018). Cell-free mitochondrial DNA in progressive multiple sclerosis. Mitochondrion.

[39] Varhaug, K. N. *et al*. Increased levels of cell-free mitochondrial DNA in the cerebrospinal fluid of patients with multiple sclerosis. *Mitochondrion*. **34**:32–35, 10.1016/j.mito.2016.1012.1003. Epub2016 Dec 1023 (2017). 10.1016/j.mito.2016.12.003 28017684

[40] Bandelt HJ, Forster P, Sykes BC, Richards MB (1995). Mitochondrial portraits of human populations using median networks. Genetics..

[41] Yarham JW (2011). A Comparative Analysis Approach to Determining the Pathogenicity of Mitochondrial tRNA Mutations. Human Mutation.

[42] Yarham, J. W., McFarland, R., Taylor, R. W. & Elson, J. L. A proposed consensus panel of organisms for determining evolutionary conservation of mt-tRNA point mutations. *Mitochondrion*. **12**, 533–538, 10.1016/j.mito.2012.1006.1009. Epub2012 Jul 1017 (2012). 10.1016/j.mito.2012.06.009 PMC3510436 22781547

[43] Weissensteiner, H. *et al*. HaploGrep 2: mitochondrial haplogroup classification in the era of high-throughput sequencing. *Nucleic Acids Res*. **44**, W58–63, 10.1093/nar/gkw1233. Epub2016 Apr 1015 (2016). 10.1093/nar/gkw233 PMC4987869 27084951

[44] Herrnstadt, C. *et al*. Reduced-median-network analysis of complete mitochondrial DNA coding-region sequences for the major African, Asian, and European haplogroups. *Am J Hum Genet*. **70**, 1152–1171. Epub 2002 Apr 1155 (2002). 10.1086/339933 PMC447592 11938495

[45] Behar DM (2012). A “Copernican” reassessment of the human mitochondrial DNA tree from its root. Am J Hum Genet..

